# The influence of spinal venous blood pressure on cerebrospinal fluid pressure

**DOI:** 10.1038/s41598-023-48334-8

**Published:** 2023-11-28

**Authors:** Z. Taylor, C. English, M. Cramberg, B. A. Young

**Affiliations:** Department of Anatomy, Kirksville College of Osteopathic Medicine, Kirksville, MO 63501 USA

**Keywords:** Neuro-vascular interactions, Neurophysiology

## Abstract

In *Alligator mississippiensis* the spinal dura is surrounded by a venous sinus; pressure waves can propagate in the spinal venous blood, and these spinal venous pressures can be transmitted to the spinal cerebrospinal fluid (CSF). This study was designed to explore pressure transfer between the spinal venous blood and the spinal CSF. At rest the cardiac-related CSF pulsations are attenuated and delayed, while the ventilatory-related pulsations are amplified as they move from the spinal venous blood to the spinal CSF. Orthostatic gradients resulted in significant alterations of both cardiac- and ventilatory-related CSF pulsations. Manual lateral oscillations of the alligator’s tail created pressure waves in the spinal CSF that propagated, with slight attenuation but no delay, to the cranial CSF. Oscillatory pressure pulsations in the spinal CSF and venous blood had little influence on the underlying ventilatory pulsations, though the same oscillatory pulsations reduced the ventilatory- and increased the cardiac-related pulsations in the cranial CSF. In *Alligator* the spinal venous anatomy creates a more complex pressure relationship between the venous and CSF systems than has been described in humans.

## Introduction

Phase-contrast MRI studies, which can quantify fluid flow, have shown that flow of the cerebrospinal fluid (CSF), particularly spinal CSF, is closely correlated to the ventilatory cycle^1,2^. Increased resolution of the MRI techniques showed that venous flow within the spinal veins matches the spinal CSF flow^3^. These MRI studies extended the older physiological studies which demonstrated that changes in intrathoracic pressure, whether by ventilation^4^ or artificially induced^5^, resulted in changes in central venous pressure and CSF pressure. Experimental studies have shown that changes in venous pressure result in changes in local dural compliance and spinal CSF pressure^6,7^. It has been hypothesized that disruption of the normal link between the venous and CSF systems is a contributor to hydrocephalus^8,9^ and multiple sclerosis^10,11^. Still, experimentally connecting spinal CSF pressures to spinal venous pressures is challenging, particularly given the size and location of the spinal venous plexus^12^.

**Figure 1:**
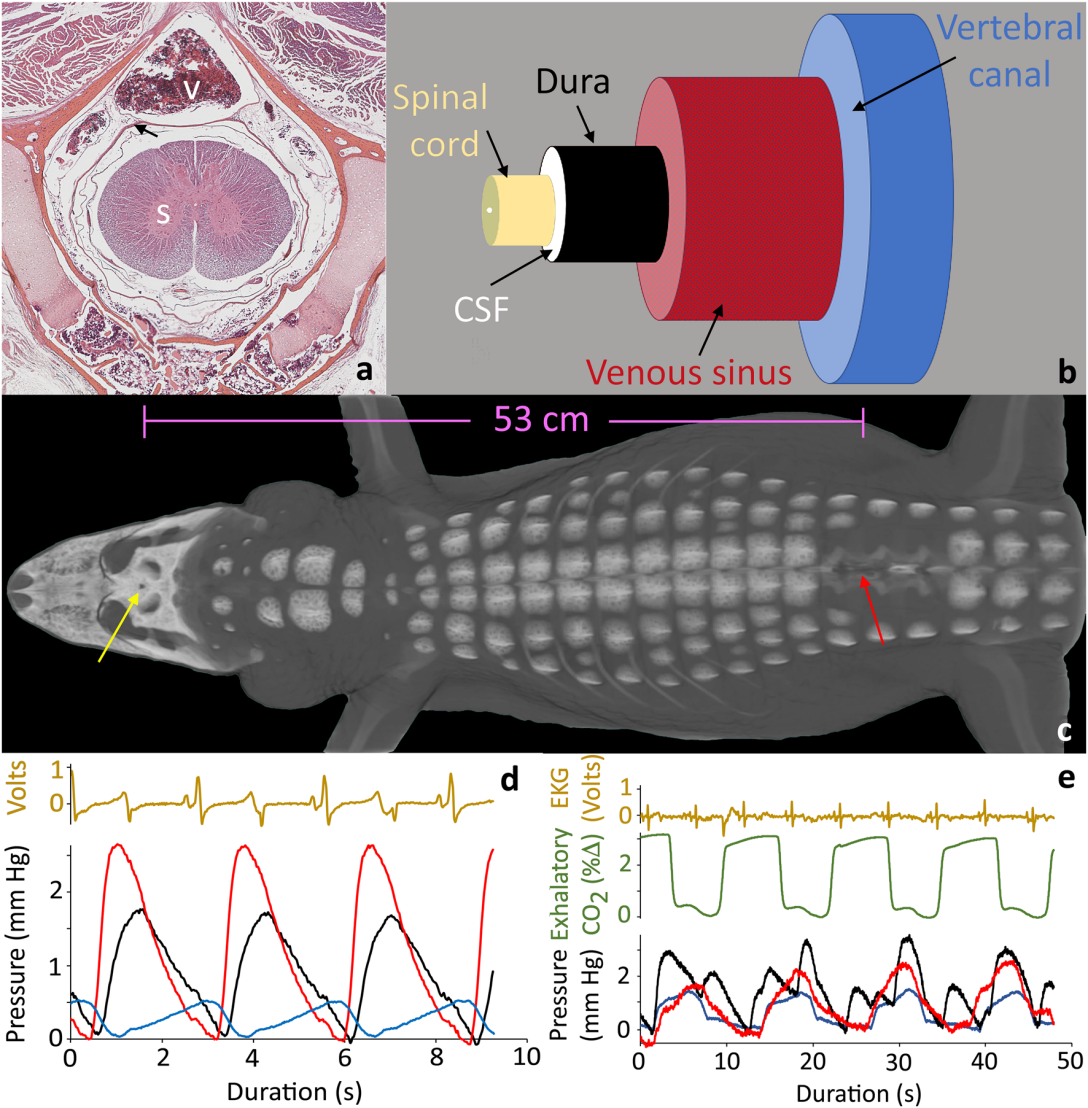
Anatomical features and baseline pressures of *Alligator mississippiensis*. (**a**) transverse section through a cervical vertebra; the dura (arrow) separates the cerebrospinal fluid (CSF) surrounding the spinal cord (s) from the larger spinal venous sinus (v). (**b**) schematic of the fluid systems examined in this study, the venous sinus fills the majority of the vertebral canal and has a cross-sectional area roughly 2.5 × that of the spinal CSF. (**c**) 3-D reconstruction of a CT scan of a 178 cm *Alligator mississippiensis*; the surgical opening used to record cranial CSF pressure (yellow arrow) was a mean of 51.6 cm from the laminectomy used to record venous and spinal CSF pressures (red arrow). (**d**) Pressure pulsations in the spinal CSF (red), cranial CSF (black), and spinal venous blood (blue) in relation to the EKG (gold) during apnea. (**e**) Baseline pressure traces in the spinal CSF (red), cranial CSF (black), and spinal venous blood (blue) in relation to the EKG (gold) and ventilatory cycle (green); the cranial CSF pressure reflects both the cardiac and ventilatory cycles, while the spinal CSF pressure and venous blood pressure are generally dominated by the ventilatory cycle.

In crocodylians, and other reptiles, the spinal cord runs the length of the body including the tail; there is no equivalent of a lumbar cistern to serve as a CSF pressure reservoir^13^. The epidural space in reptiles differs from that of humans and other mammals^14^. In *Alligator* there are no epidural fat deposits; instead, the epidural space is filled with vascular tissue (Fig. 1A,B), predominantly by the large spinal venous sinus^15^ which drains the brain and skull^16^, continues throughout the length of the trunk, and (in a reduced form) along the length of the tail. The American alligator (*Alligator mississippiensis*) has both ventilatory and cardiac pressure pulsations within the CSF^17^, similar to what has been described in humans and other mammals. Furthermore, *A. mississippiensis* has a diaphragm and is capable of generating and maintaining transdiaphragmatic pressures^18^. This suggests that a functional coupling between intrathoracic pressure, spinal venous pressure, and spinal CSF pressure is present in *A. mississippiensis*.

The presence of a venous sinus surrounding the spinal dura (Fig. 1A,B), and the continuity of that sinus along the length of the spinal cord suggests that in *Alligator*, the influence of venous pressure waves on spinal CSF may be greater than has been reported in mammals (where the venous blood is in a plexus). The present study was designed to explore the relationships between cranial CSF pressure, spinal CSF pressure, and spinal venous blood pressure in *Alligator mississippiensis*. In particular, the temporal and amplitude features of these three pressures will be examined in relation to cardiac and respiratory patterns. The relative stability of the recorded patterns will be examined by experimental perturbations of the system.

## Results

### Baseline pulsations

For this study, baseline pulsations include the ventilatory pulsations recorded under isoflurane anesthesia, and the cardiac pulsations recorded during forced apnea. The cardiac pulsations were largest in the cranial CSF and smallest in the venous blood pressure (Fig. 1D, Table 1). MANOVA yielded a F value of 105.74, and a *p* value of 1.11 × 10–16;

**Table 1:** Summary of the quantitative features of the pressure pulsations at rest and during orthostatic challenges (above) and during manual tail oscillations (below).

		Baseline		Head-up		Head-down	
		Amplitude	Temporal	Amplitude	Temporal	Amplitude	Temporal
Baseline and orthostatic pressures							
Cardiac	Cranial CSF	1.66, 0.59	0.42, 0.04			0.94, 0.35	0.26, 0.05
	Spinal CSF	1.14, 0.23	0.61, 0.09			0.66, 0.33	0.50, 0.02
	Venous blood	0.27, 0.10	0.98, 0.06			0.17, 0.04	1.04, 0.03
Ventilatory	Cranial CSF	1.45, 0.43	0.64, 0.06	2.30, 0.50	0.65, 0.06		
	Spinal CSF	1.58, 0.56	0.65, 0.06	1.82, 0.14	0.65, 0.07		
	Venous blood	1.09, 0.28	0.64, 0.07	2.03, 0.42	0.69, 0.03		

	Pressure pulsations during tail oscillation				Tail kinematics		
	Peak-to-peak	Power	Peak Amp	Peak Hz	Tail Hz	Tail Amp	
Tail oscillation experiments							
Cranial CSF	3.65, 1.70	0.45, 0.24	0.40, 0.23	4.99, 1.9	2.83, 0.29	8.13, 2.52	
Spinal CSF	4.14, 1.51	0.52, 0.14	0.48, 0.16	4.61, 1.49	2.62, 0.78	7.31, 3.06	
Venous blood	3.30, 1.25	0.39, 0.09	0.33, 0.09	5.08, 1.35	2.86, 0.28	8.42, 2.70	

All values are given in the form: mean, standard deviation. All amplitudes are in mm Hg, the temporal values are percent of the respective (cardiac or ventilator) cycle, peak-to-peak is the mean value of the pressure pulsations, while Power, Peak amplitude, and Peak Hz were all determined by power spectral analysis. Tail amplitude is given in cm.

Tukey’s Post-Hoc analysis shows that all three cardiac pulsations are significantly different in amplitude. The cardiac pulsations peaked first in the cranial compartment, then in the spinal compartment (after a delay of nearly 20% of the cardiac cycle), and lastly in the venous sinus (Fig. 1D, Table 1). MANOVA yielded a F value of 308.74, and a *p* value of 1.11 × 10–16;

Tukey’s Post-Hoc analysis shows that the timing of all three cardiac pulsations are significantly different. The cardiac pulsations took an average of 430 ms (s.d. = 240) to propagate the mean distance of 51.6 cm between the cranial and spinal recording sites (Fig. 1C).

The amplitude of the ventilatory pulsations were largest in the spinal CSF and smallest in the venous blood pressure (Fig. 1E, Table 1). MANOVA yielded a F value of 10.18, and a *p* value of 0.0001; Tukey’s Post-Hoc analysis showed that the venous blood pressure pulsations were significantly smaller, but that the two CSF pulsations were not significantly different. The ventilatory pulsations recorded from all three sites exhibited a similar temporal pattern relative to the ventilatory cycle; with peak amplitudes occurring near 65% of the ventilator cycle.

### Changes in pulsations during orthostatic gradients

**Figure 2:**
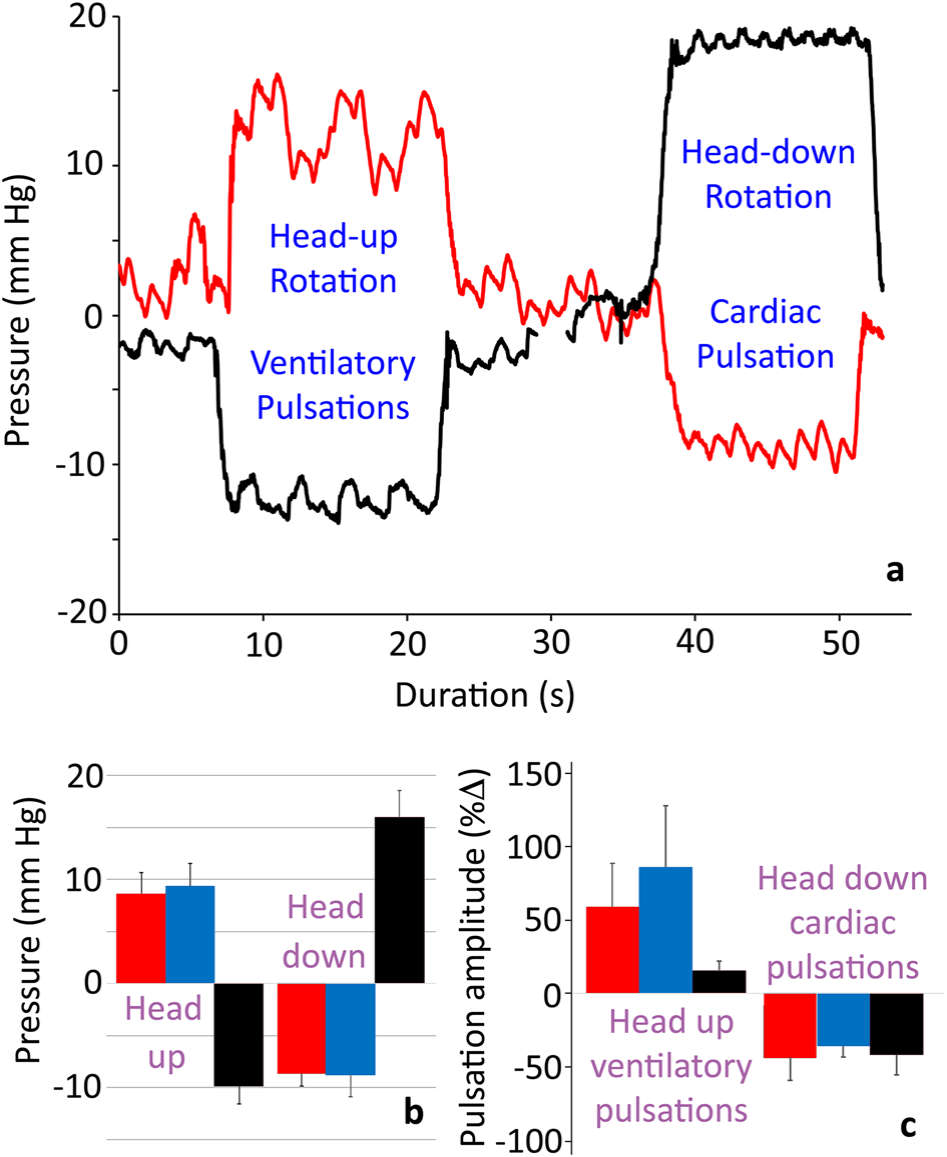
The influence of orthostatic gradients on CSF and venous blood pressure in *Alligator mississippiensis*. (**a**) Simultaneously recorded raw data traces of spinal CSF pressure (red) and cranial CSF pressure (black) during an orthostatic trial; the baseline pressures have been slightly offset for clarity. The two pressures shift in opposite directions during every rotation, but show the same “filtering” influence favoring ventilatory pulsations during head-up and cardiac pulsations during head-down rotations. The cardiac CSF shift is asymmetric (being greater during head-down rotations) while the spinal CSF shift is symmetric. (**b**) Baseline pressure shifts during the orthostatic trials; columns are the mean of the eight trials, error bars are 1 standard deviation. The spinal CSF (red) and venous blood (blue) shifts are symmetric and similar; the cranial CSF (black) shifts are significantly larger and asymmetric. (**c**) Changes in the pressure pulsations during the orthostatic trials; columns are the mean of the eight trials, error bars are 1 standard deviation. The spinal CSF (red), cranial CSF (black), and venous blood (blue) pulsations all increase in amplitude during head up rotations due to emphasis on the ventilatory influence; the pressure pulsations all decrease in amplitude during head-down rotations, due to the reduction/loss of the ventilatory pulsations. Note that the increase in pressure pulse amplitude observed during head-up rotations is significantly less in the cranial CSF.

Tilting the alligator head-up or head-down (Fig. 2A–C) produced four general patterns: (1) the spinal CSF and venous blood pressures always shifted in the opposite direction from the cranial CSF pressure; (2) the spinal CSF and venous blood pressures showed a symmetrical response to rotation, while the cranial CSF pressure change was asymmetrical with greater pressure change during head-down rotations; (3) there was no evidence of compensation within any of the measured pressures; and (4) during head-up rotations the ventilatory pulsations were more prominent in all three pressures (cardiac pulsations were often absent), while during head-down rotations cardiac pulsations were prominent in all three pressures (ventilatory pulsations were greatly reduced or absent), resulting in a “filtering” effect.

During the head-up rotations, the amplitudes of the ventilatory pulsations increased in all three pressures (Fig. 2A,C; Table 1). The pressure increases were significant in the cranial CSF (t = 4.39, *p* = 0.0001) and venous blood pressure (t = 4.79, *p* = 0.00003), but not in the spinal CSF (t = 1.03, *p* = 0.311). The temporal pattern of the ventilatory pulsations showed little to no change during the head-up rotations (Fig. 2A; Table 1). During head-down rotations the amplitudes of the cardiac pulsations decreased in all three pressures (Fig. 2A,C; Table 1), but only the decrease in spinal CSF pulsation amplitude was significant (t = 4.11, *p* = 0.0002). During head-down rotations the temporal pattern of the venous cardiac pulsations was stable, while the cranial and spinal CSF pulsations both occurred significantly (t = 12.67, *p* = < 0.00001 and t = 3.2, *p* = 0.003; respectively) earlier in the cardiac cycle (Fig. 2A; Table 1).

### Pressure changes during tail oscillations

**Figure 3:**
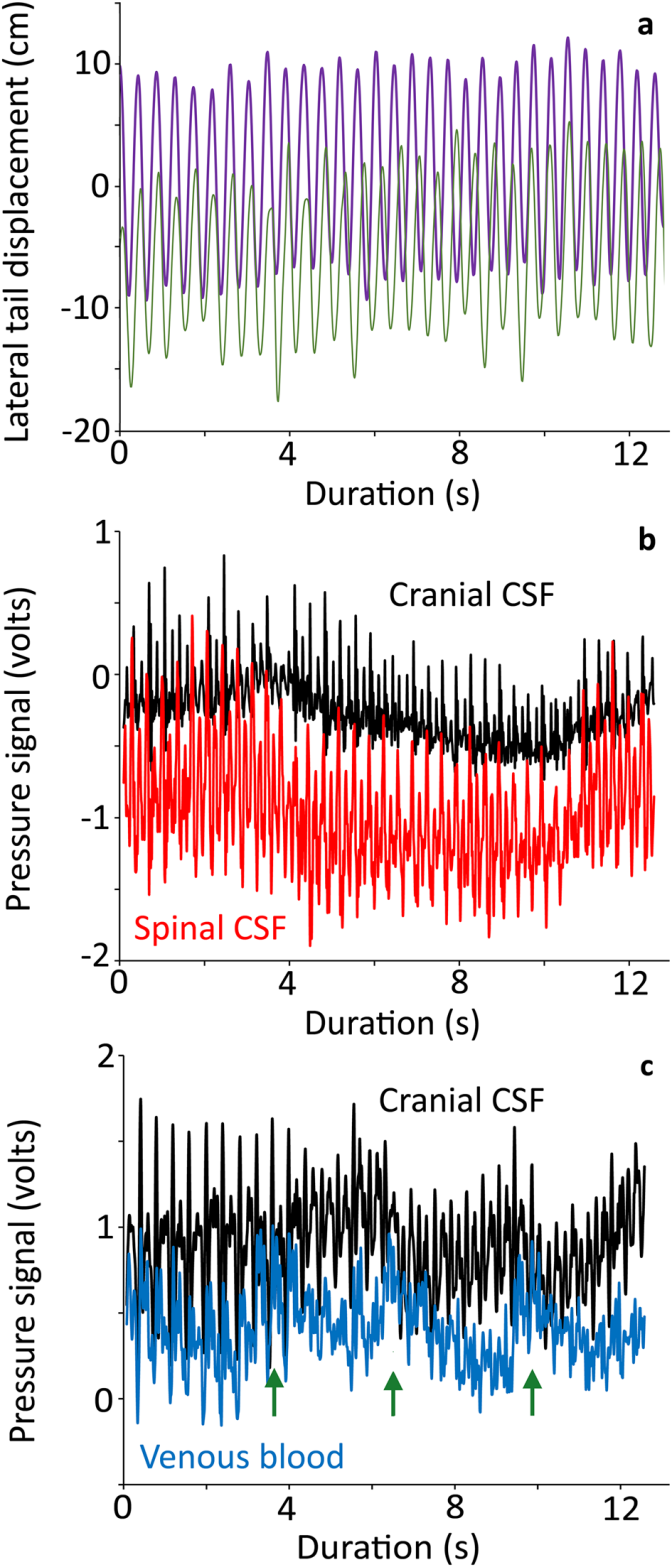
Kinematic displacement and pressure pulsations recorded during manual oscillation of the tail of *Alligator mississippiensis*. (**a**) displacement of the distal tip of the tail during two oscillatory bouts. Note that for clarity one bout of tail oscillation (thick purple trace) was left at the original resting point of 0, while the second bout (thin green trace) was adjusted to a resting point of − 10. (**b**) simultaneously recorded raw data traces of cranial CSF pressure (black) and spinal CSF pressure (red); the individual pulsations and baseline shifts are the same in the two traces. (**c**) simultaneously recorded raw data traces of cranial CSF pressure (black) and venous blood pressure (blue); the pulsations within the venous blood pressure are not as consistent as those within the cranial CSF pressure, and the baselines of the two traces diverge then converge (green arrows) at a frequency corresponding to the cardiac cycle.

During the tail oscillations, the distal tip of the tail was held (with minimal elevation off the platform) then oscillated laterally through a range of approximately 30 cm (Fig. 3A). When measured near the tail base the oscillations of the tail had a frequency range of 2.2–3.4 Hz (mean = 2.8, s.d. = 0.28), and an amplitude range of 3.65–16 cm (mean = 8.8, s.d. = 2.9). To accomplish this, the distal tip of the tail typically had mean (15.5 m/s^2^) and maximum (36 m/s^2^) accelerations. The amplitude of the tail oscillations had a negative slope when plotted against oscillation frequency.

Each bout of tail oscillation had a duration of approximately 15 s. There was a strong temporal congruence between the simultaneously recorded spinal and cranial CSF pressures traces within each oscillation bout (Fig. 3B). The baseline shifts were mirrored between the two pressure traces, and the individual pressure pulses were aligned. In both data traces (Fig. 3B) there were higher amplitude pulsations (which occur at a frequency near 3 Hz) interspersed with lower amplitude pulsation (the total pulsations have a frequency near 6 Hz). A different pattern was present between the simultaneously recorded venous blood pressure and cranial CSF pressure within each oscillation bout (Fig. 3C). The pulsation pattern was not as consistent in the venous blood pressure. The baseline pressures diverged (slowly) then converged (more rapidly) repeating approximately every 3.5 s (0.28 Hz), Despite the greater irregularity in the venous pressure pulsations, overall there was a strong temporal agreement between the two data channels (Supplementary Information 1).

**Figure 4:**
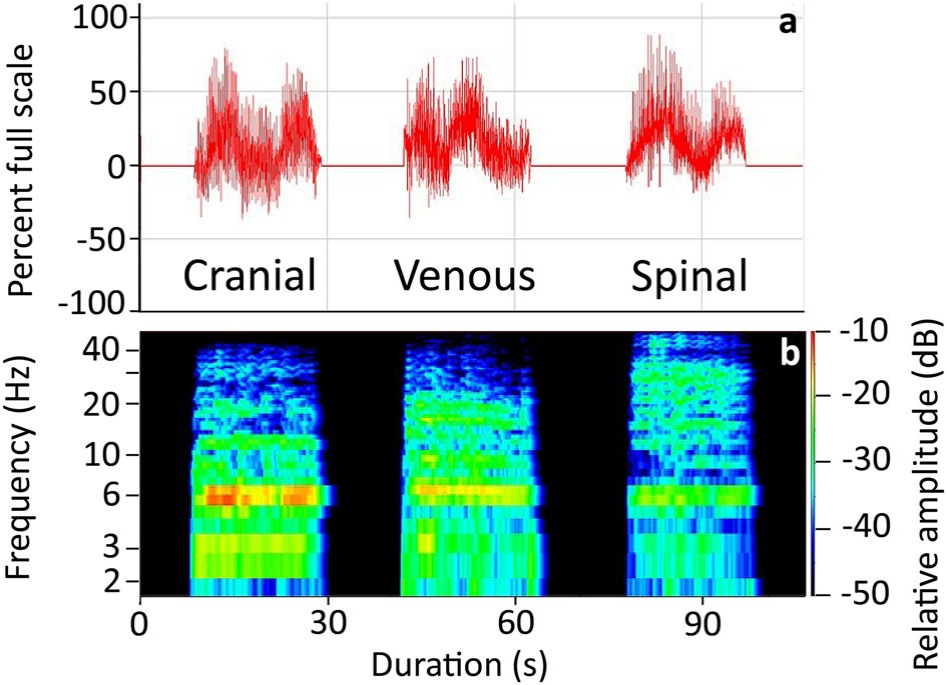
Frequency analysis of the pressures recorded during bouts of manual oscillation of the tail of *Alligator mississippiensis*. (**a**) power graphs of the three pressure curves look similar reflecting the relative consistency of the tail oscillations across trials. (**b**) spectral analysis reveals a similar presence of a dominant frequency near 3 Hz (the oscillatory frequency of the tail) and a harmonic near 6 Hz; the spinal CSF pressures had more developed higher-order harmonics, while the cranial CSF pressure had prominent low-frequency components but little higher-order energy.

Spectral analysis of the tail oscillation pressure waves (Fig. 4) revealed that all three pressures (cranial CSF, spinal CSF, and venous blood) had similar spectral patterns. There was a frequency band near 3 Hz (corresponding to the oscillation frequency of the tail), then a first harmonic (near 6 Hz) which was often the dominant frequency. Beyond these similarities, there was an interesting pattern in the spectra. The spectra of the spinal CSF pressure during tail oscillations generally had a less prominent dominant frequency, and more prominent higher harmonics (Fig. 4). The spectra of the cranial CSF pressures during tail oscillations had the most prominent dominant frequency, but little higher-order harmonics. The spectral analysis of the venous blood pressure during the tail oscillations was intermediate between the two CSF pressures (Fig. 4).

**Figure 5:**
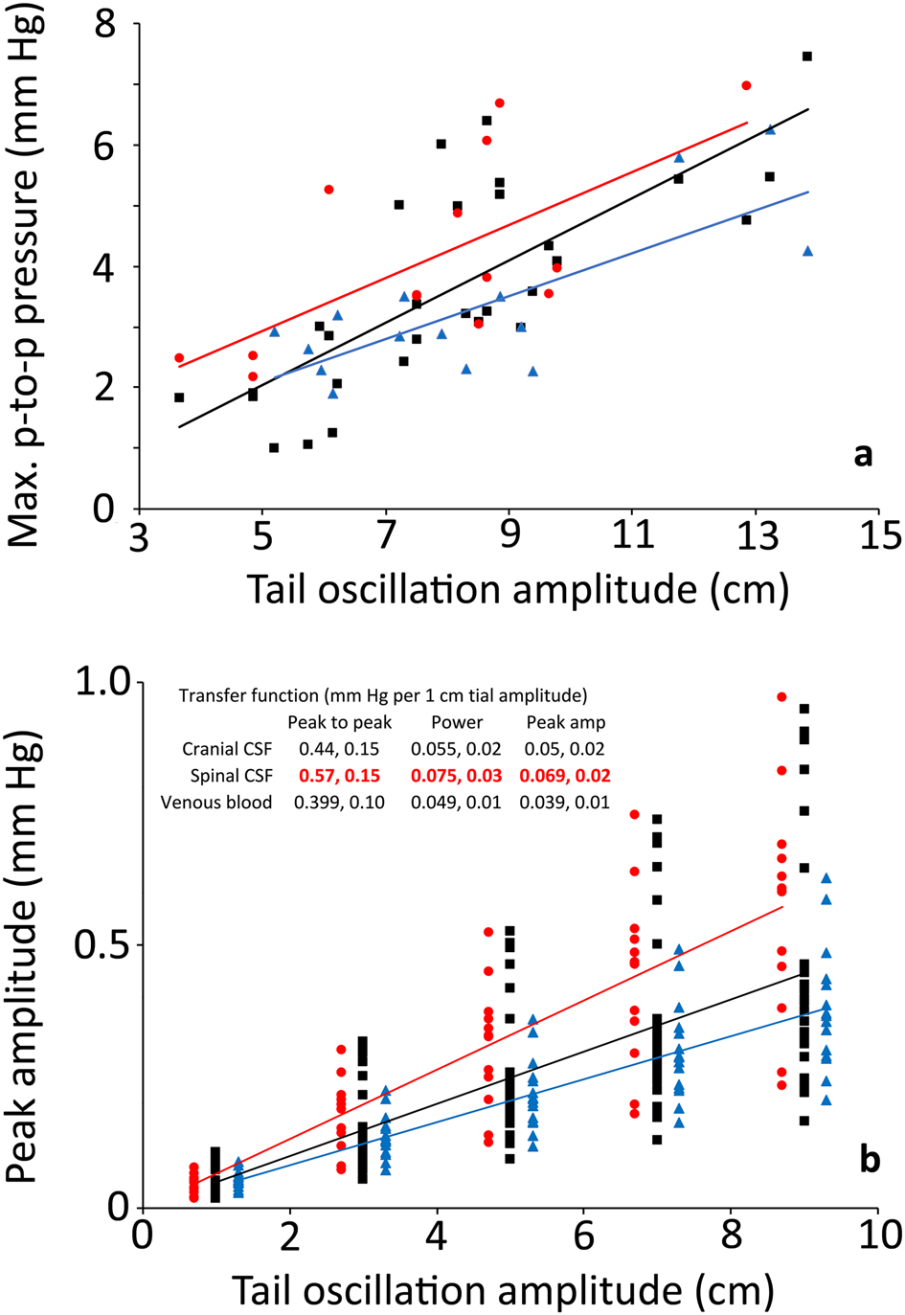
Relationship between the kinematics of tail oscillation and the recorded pressures in *Alligator mississippiensis*. (**a**) the spinal CSF (red), cranial CSF (black), and venous blood (blue) pressures all increased with increasing amplitudes of tail oscillation. Note that there were no significant differences among the slopes of the three lines. (**b**) transfer functions (mm Hg change per 1 cm tail oscillation, given as mean, s.d.) were calculated based on the experimental data (inset), the transfer functions for the spinal CSF were significantly greater than for the cranial CSF or venous blood pressure. When the transfer functions from each animal are graphed, the spinal CSF values (red) are distinct from the cranial CSF (black) or venous blood pressure (blue).

Temporal and amplitude analyses of the different pressure responses to tail oscillation, revealed considerable overlap between the cranial CSF, spinal CSF, and venous blood pressures (Table 1). While there was a clear trend in the pressure amplitudes (spinal is always largest and venous always smallest), MANOVA performed with a Bonferroni-adjusted significance level revealed no significant differences among the amplitude or temporal features. In part, this may simply reflect that the oscillations used to produce these pressures had a range of kinematic features. If pressure amplitude was plotted against tail amplitude (Fig. 5A), there was a clear divergence between the spinal CSF and venous blood pressure, suggesting that larger movements of the tail would result in significant differences among the pressures. Another way of looking at this was to determine a form of transfer function, essentially the increase in pressure produced by each cm of tail oscillation. In these transfer functions (inset, Fig. 5B) the spinal CSF values were significantly larger than the other two (as assayed by MANOVA and post-hoc Tukey’s test). A transfer function was determined for each tail oscillation bout and these were used to plot model response curves (Fig. 5B), the slope of the response was the average mm Hg/1 cm tail oscillation given in the insert. All of these analyses indicated that the amplitude of tail oscillation had the greatest influence on spinal CSF pressure; while not significantly different, the venous blood pressure had a consistently lower response to the amplitude of tail movement than did the cranial CSF pressure.

The frequency analysis of these pressure was less conclusive, primarily because all three pressures (cranial CSF, spinal CSF, and venous blood) had a fundamental frequency near 3 Hz that corresponded to the oscillatory frequency of the tail (Table 1) and a harmonic near 6 Hz that typically formed the dominant frequency (Fig. 4). This bimodal distribution (between the fundamental frequency and the first harmonic) was present in all three pressures and results in the large standard deviations in the frequency of maximum pressure (Table 1).

**Figure 6:**
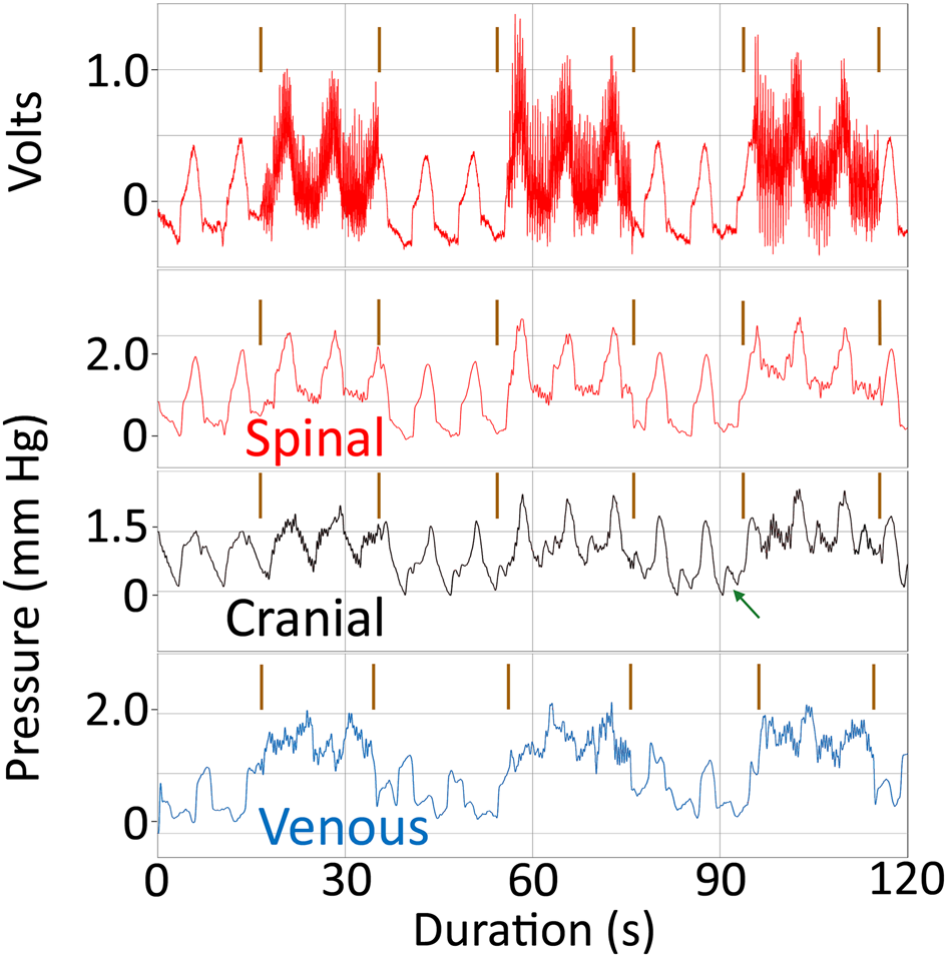
Influence of tail oscillations on the underlying pressure pulsations. Top trace is raw data from the spinal CSF pressure showing the three bouts of tail oscillation (vertical lines). Immediately below is the same data record after the filtering; note that the periods of tail oscillation caused an increased in baseline pressure, but little change to the ventilatory pulsations. The cranial CSF (black) and venous blood pressure (blue) traces were all recorded from the same animal within a few minutes of the spinal CSF record; all three pressure traces were subjected to the same filtering. The tail oscillation raises the baseline pressure of the spinal venous blood, and disrupts the pattern of the ventilatory pulsations. In the cranial CSF pressure, the tail oscillations raised the baseline pressure, but also fundamentally changed the shape of the underlying pulsations reducing the time of the ventilatory pulsations and increasing the prominence of cardiac pulsations (arrow).

**Figure 7:**
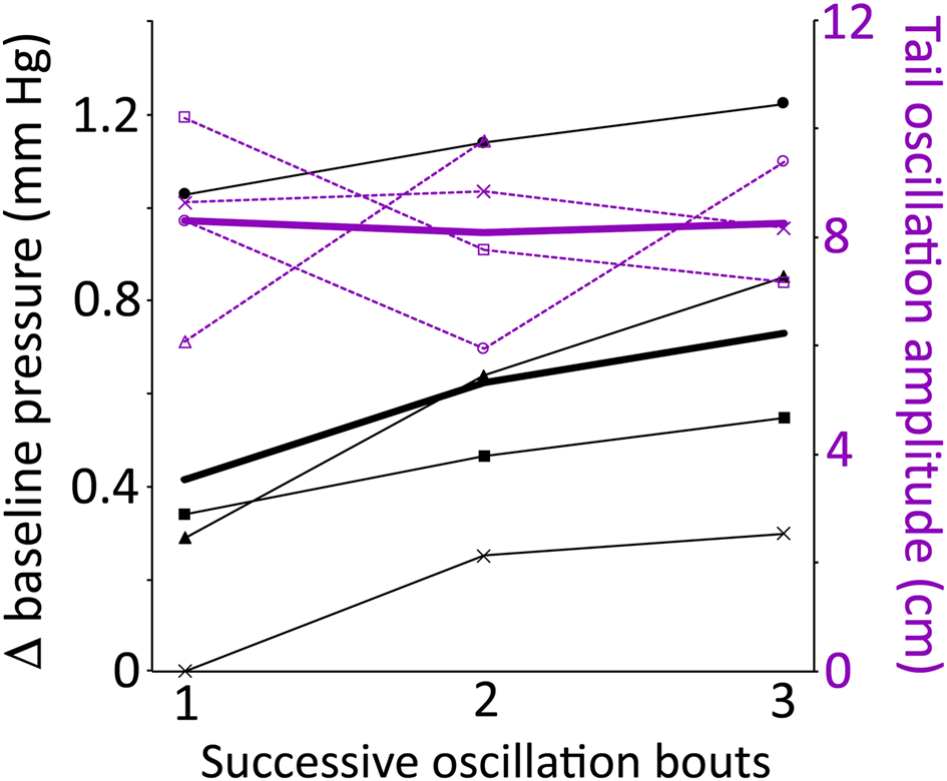
Relationship between tail oscillation and increases in baseline pressure. All of the recorded pressures increased in baseline pressure during tail oscillation, and this increase was typically incremental with greater baseline shifts with successive bouts of tail oscillation (Fig. 6). If the magnitude of the cranial CSF baseline shift of four specimens (thin black lines) is plotted across the three bouts of tail oscillation, each of the four pressure traces increases, as does the mean (thick black line). If the tail oscillation amplitude (purple dashed lines) are plotted for the same specimens (specimens are symbol coded) there is no clear pattern of increasing tail amplitude across the three bouts of tail oscillation (mean value is the thick purple line). This indicates that the shift in pressure baseline is not driven solely by the oscillation of the tail.

To look at the impact of tail oscillations on the underlying interactions and stability of the cranial CSF, spinal CSF, and venous blood pressures a digital filter was constructed (2 Hz low pass filter with 5000 taps and a gain of − 0.25 dB) and applied to all of the raw tail oscillation pressure records (Supplementary Information 1). This filter was designed to remove the influence of the oscillations, while having minimal influence on the ventilatory and cardiac pulsations (which occurred at frequencies well below the 2 Hz margin of the low-pass filter). With the oscillatory pulses filtered out (Fig. 6), it was clear that the onset of the oscillations was associated with an increase in baseline pressure. This increase was greatest in the spinal CSF (mean = 0.71 mm Hg, s.d. = 0.4) and lowest in the venous pressure (mean = 0.44 mm Hg, s.d. = 0.16); MANOVA found no significant differences in the magnitude of this oscillatory baseline shift among the three pressures. The magnitude of the baseline shift was not constant; consistently in the cranial CSF (and in roughly half of the trials in the other pressures) the shift in the baseline was incremental (Fig. 7). With each successive oscillation bout the baseline increased further, despite no corresponding pattern in the amplitude of the tail vibrations (Figs. 6 and 7).

The pressure trace from the venous sinus (Fig. 6) showed a steady pattern of ventilatory pulsations, periodically interrupted by the tail oscillations. The ventilatory pulsations were present during the tail oscillations, but they were reduced in amplitude and consistency. The spinal CSF traces showed a steady pattern of ventilatory pulsations. With the exception of the increased baseline, there was little evidence in the spinal CSF trace of any perturbation; though during the third consecutive oscillatory bout (Fig. 6) the ventilatory pulsations were slightly reduced in amplitude and duration. Tail oscillations produced the largest changes in the cranial CSF pressure traces. With repeated bouts of tail oscillation, the ventilatory pulsations underwent progressive reduction of pulse duration with a slight increase in pulse amplitude, and cardiac pulsations became evident between successive ventilatory pulses. These cardiac pulsations were evident between the periods of tail oscillation (green arrow on Fig. 6) as well as during the tail oscillations (especially the latter oscillations in the series).

## Discussion

Recordings from the spinal venous sinus reveal that the blood pressure is actively shifting, reflecting the physiological and physical influences on the animal, and that the shifts in sinus blood pressure have a significant impact on the magnitude and propagation of the CSF pressure.

With the alligator in a resting anesthetized state, the spinal venous sinus has two, almost contradictory, influences on the CSF pressure. On the one hand, it attenuates and delays the cardiac-related pulsations; on the other hand, it transmits the ventilatory pressure pulsations to the CSF. The cardiac pulsations recorded from the spinal CSF are significantly lower in amplitude than the cranial cardiac pulsation (Fig. 2, Table 1) and take a mean of 430 ms to propagate from the cranial to the spinal recording site; propagation delays between cranial and spinal CSF pressure recording sites have been previously reported^19^. Studies have shown that in some mammals the cardiac pulsations recorded in the spinal CSF originate, in part at least, from arterial pulsations within the spinal cord^20,21^. Given that the spinal and cranial recording sites were equidistant from the heart, a spinal origin for the cardiac CSF pulsation would be difficult to reconcile with the recorded propagation delay.

*Alligator mississippiensis* has a functional diaphragm, often termed the postpulmonary septum, that is capable of creating and maintaining transdiaphragmatic pressures^18^. The active ventilation applied during surgery would have created cyclic changes in intrathoracic pressures^18^, which, in turn, would have produced changes in central venous pressure and, ultimately, spinal sinus pressures, in the same manner that has been documented in man and other mammals^22^. In the alligator, the ventilatory pulsations in the venous system result in cranial CSF pulsations very similar to what has been detailed in humans^23,24^. In the spinal CSF, where the venous sinus has a cross-sectional area some 2.5 times greater than the subdural space (Fig. 1A,B) the size differential creates a fluid pressure amplifier. This results in nearly simultaneous ventilatory pressure pulsations (Table 1) in the venous sinus, cranial CSF, and spinal CSF. The CSF ventilatory pulsations were significantly larger than those in the venous blood pressure.

When the alligator was placed in the head-up position, the blood pressure in the spinal sinus increased (Fig. 2) presumably due to venous blood pooling caudal to the heart. The CSF pressure increased in the spinal compartment, but decreased in the cranial compartment, because the hydrostatic indifferent point of the alligator is near the heart, as it is in humans and other mammals^25^. The caudal pooling of the venous blood appears to have decreased cardiac output, and with it cranial perfusion. This shift, combined with a decrease in cranial CSF pressure, greatly reduced and/or eliminated the cardiac pulsations from the CSF^26^. The caudal pooling of venous blood, likely combined with a caudal shift in the peritoneal viscera, changed the balance between intrathoracic pressure and central venous pressure^27,28^, leading to increased ventilatory pulsations in the venous blood, and the CSF (Fig. 2).

With the alligator in a head-down position, venous blood would pool in the rostral half of the body; decreasing the venous blood pressure recorded near the pelvis (Fig. 2). This shift in blood distribution would likely increase cardiac output^29^. The increased cardiac output, perhaps coupled with a slight gravitational acceleration, significantly reduced the time (Fig. 2A) to peak cardiac CSF pressure^30^. At the same time, the combination of rostral pooling of the blood and rostral pooling of the CSF, dramatically raised intracranial pressure (Fig. 2) which significantly damped the amplitude of the cardiac CSF pulsations (Fig. 2A,C) as it does in humans^31^. With the alligator in the head-down position, a slight shift in the peritoneal viscera would have raised intrathoracic pressure, which, when combined with the increase in central venous pressure, would have damped the ventilatory pulsations^32^.

Manual oscillation of the anesthetized alligator’s tail produced two interacting results; the oscillatory pressure pulsations, and changes to the underlying baseline pressures. The oscillatory pressure pulsations were recorded in the spinal CSF, cranial CSF, and venous sinus blood. The pulsations were rather consistent in these three sources (Fig. 4, Table 1). The oscillatory pressure pulsations were consistently largest in the spinal CSF (Fig. 5, Table 1) and were then damped as the pulsations propagated rostrally. This damping or attenuation is evident in both the lower magnitude of the cranial oscillatory pulsations (Table 1), and the loss of the higher-order harmonics (Fig. 4) between the spinal and cranial CSF^33^. Unlike the attenuation of the caudally-propagating cardiac pulsation, the attenuation of the rostrally-propagating oscillatory pulsations do not involve any delay. The oscillatory spinal CSF and cranial CSF pulsations are synchronized (Fig. 3A). The source(s) for the oscillatory pressure pulses in the spinal venous blood pressure are not clear. Herein it is hypothesized that the oscillatory pulses are shaped by a combination of three sources: (1) directly by the manual oscillations of the tail (acting on the spinal venous sinus of the tail and/or the interconnected^15^ caudal vein; (2) CSF pressure pulsations transmitted via the spinal dura; and (3) there appear to be physiological interactions in that the 0.28 Hz nodal oscillations present in the simultaneously recorded cranial CSF and venous blood pressure curves (Fig. 3B) are much slower than the tail oscillations (mean of 2.8 Hz), much faster than the ventilator cycle (mean of 0.14 Hz), but are in good agreement with the cardiac frequency (mean of 0.27 Hz). Studies in other systems have shown a coupling between oscillatory movements and cyclic changes in fluid pressure^34^; but we are unaware of previous studies looking at the interactions of oscillatory body displacement and physiological cycles (e.g., ventilation and the cardiac cycle) in respect to fluid pressure dynamics.

The oscillatory movement of the tail increased the baseline spinal CSF pressure, often incrementally, but the only other impact was a slight reduction in the ventilatory pulsations, and this was only evident toward the end of the oscillatory trials (Fig. 6). The oscillatory movement of the tail increased the baseline spinal sinus venous blood pressure, often incrementally; it also caused a reduction in the ventilatory pulsations, a reduction that was present during every oscillatory trial (Fig. 6). Tail oscillations had the largest impact on the baseline cranial CSF patterns; tail oscillations raised the baseline pressures incrementally (Fig. 7), reduced the duration and amplitude of the ventilatory pulsations (Fig. 6), and enhanced the amplitude of the cardiac pulsations (Fig. 6). The cardiac pulsations that emerge in the cranial CSF following tail oscillation do not propagate to the spinal CSF; this may simply reflect that these relatively low amplitude cardiac pulsations are “fully” attenuated. There was no significant difference between the three pressure sources in the increase in baseline pressure with tail oscillation. In between the oscillatory pulsations the spinal pressure returned to the same level, the cranial pressure baseline decreased slightly (0.1 mm Hg), while the spinal venous blood pressure increased slightly (0.1 mm Hg).

Unlike the orthostatic gradients, tail oscillations produced the same direction (and similar magnitudes) of baseline shift in all three pressures. Herein we hypothesize that tail oscillations caused a slight rostral displacement of the peritoneal viscera. Visceral displacement, particularly of the liver, is well-known in crocodylians and serves as a ventilatory mechanism^35^. This rostral visceral shift would cause an increase in intrathoracic pressure^18^, which would alter the relationship between the administered gas (which was nearly constant) and the central venous pressure^36,37^. Ultimately, we hypothesize, that the change in intrathoracic pressure would combine with the increased venous blood pressure during the tail oscillations to alter the ventilatory pulsations in all three pressure tracings. The tail oscillations trials produced similar shifts (decrease in ventilator pulsations, presence of smaller cardiac pulsations) to what were seen during the head-down orthostatic trials. Alternatively, the baseline shifts maybe a reflection of fluid inertia in the CSF and spinal venous blood; previous work has shown the importance of fluid inertia in CSF dynamics^38^.

This study was undertaken to explore the influence on cranial and spinal CSF pressures of the large spinal venous sinus of *Alligator mississippiensis*. The results demonstrate the sinus is both the transmission agent of the ventilatory CSF pressure pulsations, but also a major impediment to the propagation of the cardiac CSF pressure pulsations. The influence of the spinal venous sinus was not static; there was a clear continuum from head-up orthostatic trails, to baseline, to tail oscillations, to head-down orthostatic trials. The data suggest that this continuum is present within a fairly modest pressure range of approximately 10 mm Hg. Presumably this continuum of influence is driven predominantly by changes in spinal compliance due to changes in blood pressure within the spinal venous sinus; vascular pressures are the main determinants of compliance in the skull and vertebral canal^6^. Previous studies on *Alligator mississippiensis* have shown that CSF pressure is influenced by movement^39,40^, and that movement-related CSF pulsations asymmetrically propagate across the foramen magnum, presumably due to differential compliance^38^. The present study suggests that the spinal venous sinus plays a key role in modulating the compliance of the spinal dura, and, in this way, can significantly influence the exchange of CSF between the cranial and spinal compartments, and the basic parameters of the CSF pressure wave. The marked morphological differences in the spinal venous systems of *Alligator* and humans, makes it unlikely that this same venous-based spinal dural compliance is present in man. At the same time, it makes *Alligator* an attractive model organism with which to investigate how the CSF dynamics are influenced by movement, cardiac and ventilatory pulsations, as well as dynamic shifts in compliance.

## Materials and methods

### Live animals

Eight live sub-adult (150–187 cm total length, 15.7–20.9 kg mass) American alligators (*Alligator mississippiensis*) were obtained from the Louisiana Department of Wildlife and Fisheries. The animals were housed communally in a 29 m^2^ facility that featured three submerging ponds, natural light, and artificial lights on a 12:12 cycle. The facility was maintained at 30–33 °C; warm water rain showers were provided every 20 min, which helped maintain the facility at > 75% relative humidity. The alligators were maintained on a diet of previously frozen adult rats. When the individual animals were removed from the enclosure, they were caught by noosing, then their jaws were taped using vinyl tape. The husbandry and use of the live alligators followed all applicable federal guidelines, and were approved by the IACUC of A.T. Still University (Protocol #226, approved 16 March 2022). The manuscript was prepared in accordance with the ARRIVE guidelines.

### Experimental approach

When the individual alligator was noosed for the surgical experiment is was induced to bite a bite pad, and the animal’s mouth was taped shut around the bite pad. Each individual alligator was placed on a stiff board (244 × 28 × 3.8 cm thick), which exceeded the maximum width and length of the alligators used for this study. Six 2.5 cm wide heavy duty straps were used to secure the alligator to the board; the straps were tight enough to minimize movement of the animal but not tight enough to impede ventilation or circulation. With the alligator’s mouth held open by the bite pad, a laryngoscope was used to depress the gular valve and expose the glottis. A cuffed endotracheal tube was inserted into the larynx and connected to a custom anesthesia system that included a ventilator pump, Vaporstick anesthesia machine (Surgivet), isoflurane vaporizer (Surgivet), and Capnomac Ultima respiratory gas monitor (Datex-Engstrom). The alligators were maintained on a steady ventilatory pattern of 6–7 breaths per minute each with a tidal volume of 500 ml. Anesthesia was accomplished using 5% isoflurane. Two silver chloride surface cup electrodes, coated with a layer of conducting gel, were placed on the lateral surface of the animal, on either side of the heart. Meloxicam (at 0.2 mg/kg) was administered into the left triceps to serve as an analgesic.

A surgical drill was used to bore a 4 mm diameter hole through the dorsum of the alligator’s skull to expose the dura. A small incision was made in the dura to allow the passage of a pressure catheter. Surgical adhesive was used to seal the dura around the catheter, then epoxy cement was added to fill the surgical opening and secure the catheter to the skull. A fluid pressure transducer was mounted on the same board, at the level of the dorsum of the alligator's head. The pressure transducer, and the attached pressure catheter, were filled with a reptilian Ringers solution^41^. The pressure transducer was coupled to a strain gauge amplifier (P122, GRASS Instruments), while the EKG electrodes were connected to a DC preamplifier (P511, GRASS). The outputs from these two amplifiers were sampled at 4 kHz, simultaneously with the carbon dioxide concentration from the respiratory gas monitor, using the MiDAS (Xcitex Inc., Woburn, MA) data acquisition system.

A series of cranial CSF recordings were taken before any other invasive procedure was performed. Subsequently, laminectomies were performed on the alligator equivalent of the L2 and L3 vertebrae (Fig. 1C) in order to expose a length of the spinal venous sinus. The location of the laminectomies was chosen for two reasons: (1) it placed the spinal and cranial recording sites nearly equidistant from the heart; and (2) it placed the spinal recording site at nearly 50% the length of the spinal cord, which continues to the tip of the alligator’s tail^13^. A pressure catheter was inserted into the venous sinus and a series of simultaneous venous pressure and cranial CSF pressure recordings taken. Subsequently, the venous pressure catheter was withdrawn and a short (< 1 cm) incision made in the dorsal surface of the venous sinus. Hematostatic powder (Surgicel, Ethicon) was inserted through the incision to prevent blood flow along the dorsal surface of the spinal dura (Fig. 1C). Care was taken not to fully occlude the venous sinus with the hematostatic powder. Once the powder had “set” a surgical exposure was made through the middle of the hematostatic powder to expose the dorsal surface of the spinal dura. Once the spinal dura was exposed, and was free of venous blood, a pressure catheter was inserted into the spinal subdural space and a series of simultaneous cranial and spinal CSF pressure recordings were taken.

During each series of recordings, the pressures were recorded simultaneously with the EKG and ventilatory cycle during: (1) rest; (2) apnea; (3) orthostatic gradients; and (4) manual oscillations of the alligator’s tail. Any movements induced in the experimental preparation were recorded using digital video cameras; the records were then imported into Kinovea (kinovea.org) which was used to define landmark points on the tail which were tracked (and quantified) throughout the video sequence.

### Data analysis

For each of the experimental manipulations listed above, the temporal pattern of the three pressures (cranial CSF, spinal CSF, venous blood) were quantified relative to the cardiac and respiratory cycles. This was necessary because variation in the size of the specimens, response to the anesthesia, and duration of the experiments, led to variation in absolute duration of the cardiac cycle. When analyzed at the temporal midpoint of the experiments the eight alligators had a mean heart rate of 16.3 bpm, with corresponding cardiac cycles ranging from 2.3 to 5.6 s; the mean duration of the cardiac cycle was 4.0 s (s.d. = 1.2).

Frequency analyses (FFT and Power Spectral Analysis), of both the kinematic and physiological data, were performed with SpectraPlus (Pioneer Hill Software). The amplitude of each pressure pulsation was quantified (using MIDaS). Comparisons of the frequency, amplitude, and temporal data were conducted with MANOVA and a post-hoc Tukey’s test. Pulse markers were used to synchronize the kinematic and pressure recordings. The pressure data were resampled to match the (slower) frame rate of the video camera, then the two data traces aligned. The mathematical relationship between the instantaneous tail displacement and instantaneous pressure value (averaged over the full duration of the tail oscillation bout) is referred to as the transfer function.

### Ethics approval

The husbandry and use of the live alligators followed all applicable federal guidelines, and were approved by the IACUC of the Kirksville College of Osteopathic Medicine (Protocol #226, approved 16 March 2022). The manuscript was prepared in accordance with the ARRIVE guidelines.

### Declaration on human subjects

No human subjects were used in this study.

### Supplementary Information

Supplementary Information 1.

Supplementary Video 1.

Supplementary Information 2.

Supplementary Information 3.

Supplementary Information 4.

Supplementary Information 5.

Supplementary Video 2.

Supplementary Information 6.

Supplementary Information 7.

Supplementary Information 8.

Supplementary Information 9.

Supplementary Information 10.

## Data Availability

Data sequences, both digital video and digital physiological recordings, have been archived at Morphosource. Copies of the video and physiological recordings are available from the corresponding author at: byoung@atsu.edu.
