# Supplementary material for: The influence of spinal venous blood pressure on cerebrospinal fluid pressure
Source: Sci Rep. 2023 Nov 28;13:20989. doi: 10.1038/s41598-023-48334-8 (PMC10684553; doi:10.1038/s41598-023-48334-8)

Left Channel
Sampling Rate: 4000
FFT size: 128
Averaging: 4
Window: Hanning

Printed By: SpectraPLUS-SC Sound Card Edition
Licensed To: Anatomy
Wed Feb 22 05:08:26 2023

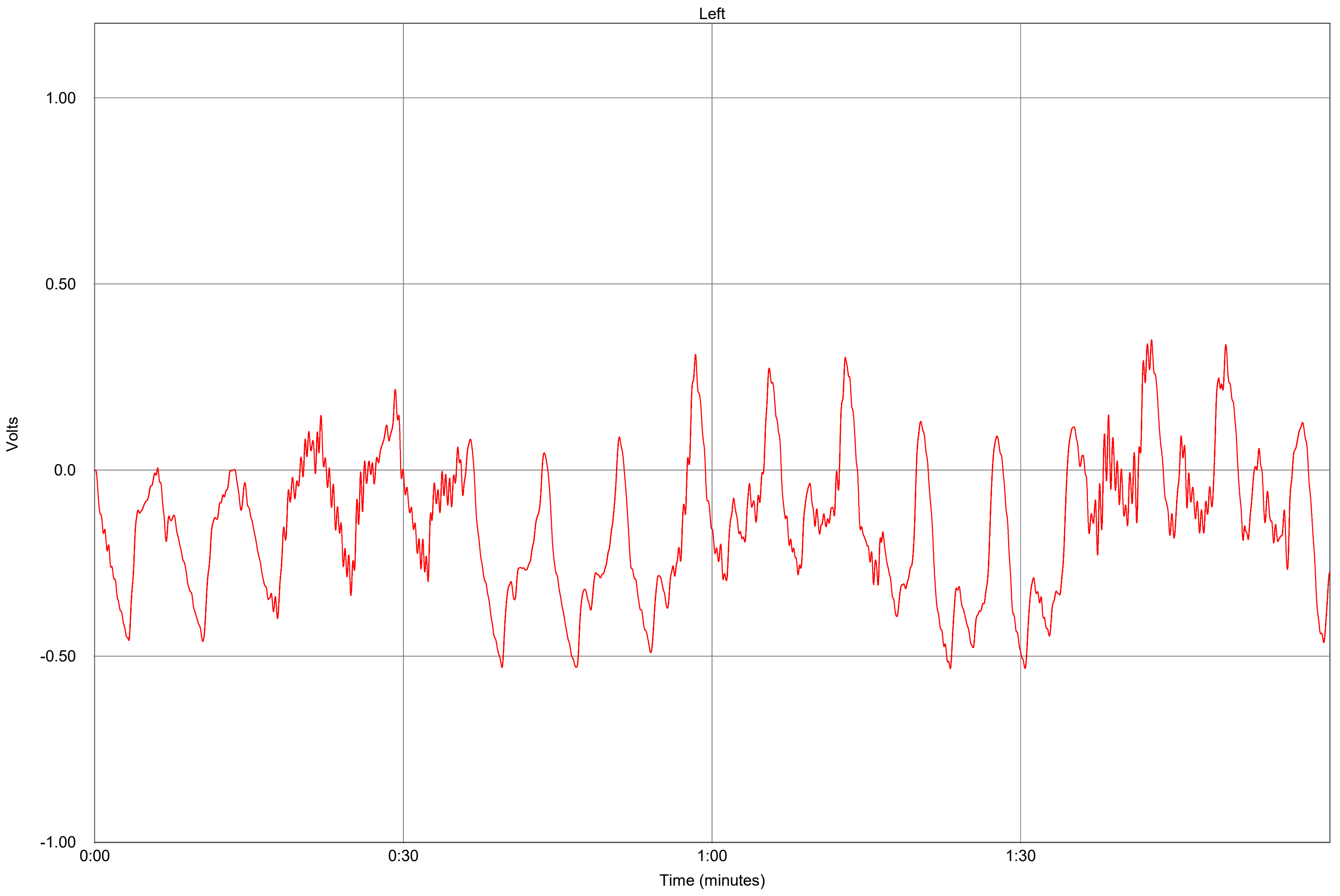

Supplement: Supplementary file 6 — Supplementary Information 5. [file 41598_2023_48334_MOESM6_ESM.pdf]
