# Supplementary material for: The influence of spinal venous blood pressure on cerebrospinal fluid pressure
Source: Sci Rep. 2023 Nov 28;13:20989. doi: 10.1038/s41598-023-48334-8 (PMC10684553; doi:10.1038/s41598-023-48334-8)
Supplement: Supplementary file 11 — Supplementary Information 9. [file 41598_2023_48334_MOESM11_ESM.docx]

Guide to the Supplemental Material

The supplemental material is all taken from an experiment performed on 12 October 2022 on a single sub-adult *Alligator mississippiensis*.

Simultaneous recording of cranial CSF pressure and spinal venous blood pressure during tail oscillation

721.mp4 is a raw video record of this trial

Supplemental screen shots (page 1) is a screen shot of the raw data on the data acquisition computer

Gator 7 file 21.xlsx is an EXCEL file of the raw data; the venous blood pressure was recorded on channel 3

721 chan 3 raw.pdf is a print out of the raw venous blood pressure traces

721 chan 3 filtered.pdf is a print out of the same venous blood pressure traces after digital filtering

Simultaneous recording of cranial CSF pressure and spinal CSF pressure during tail oscillation

731.mp4 is a raw video record of this trial

Supplemental screen shots (page 2) is a screen shot of the raw data on the data acquisition computer

Gator 7 file 31.xlsx is an EXCEL file of the raw data; the spinal CSF was recorded on channel 3, the cranial CSF on channel 5

731 chan 3 raw.pdf is a print out of the raw spinal CSF pressure traces

731 chan 3 filtered.pdf is a print out of the same spinal CSF pressure traces after digital filtering.

731 chan 5 raw.pdf is a print out of the raw cranial CSF pressure traces

731 chan 5 filtered.pdf is a print out of the same cranial CSF pressure traces after digital filtering.
