# Supplementary material for: The influence of spinal venous blood pressure on cerebrospinal fluid pressure
Source: Sci Rep. 2023 Nov 28;13:20989. doi: 10.1038/s41598-023-48334-8 (PMC10684553; doi:10.1038/s41598-023-48334-8)
Supplement: Supplementary file 12 — Supplementary Information 10. [file 41598_2023_48334_MOESM12_ESM.pdf]

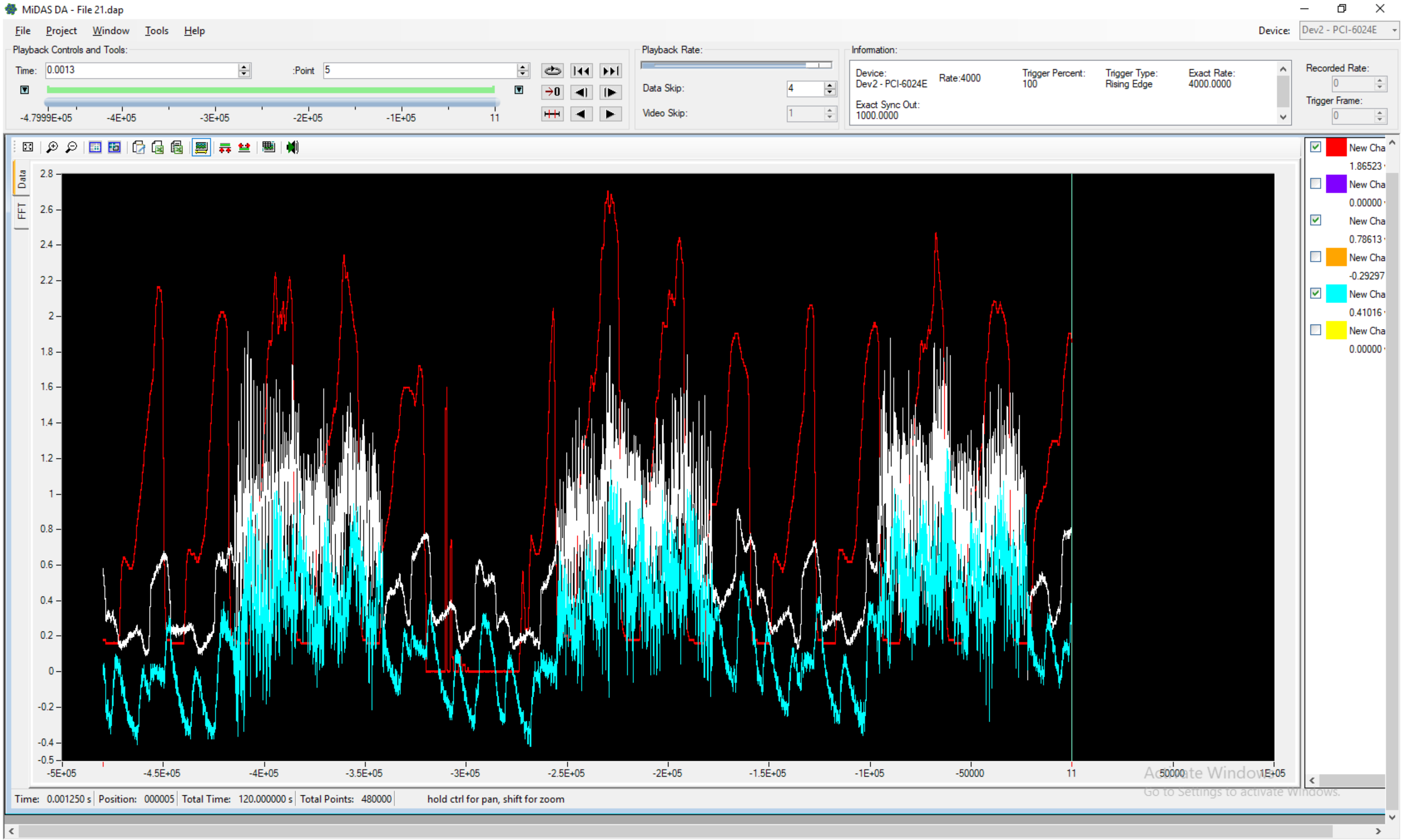
MiDAS DA - File 21.dap
File
Project
Window
Tools
Help
Device:
Dev2 - PCI-6024E
Playback Controls and Tools:
Time: 0.0013
:Point 5
-4.7999E+05
-4E+05
-3E+05
-2E+05
-1E+05
11
Playback Rate:
Data Skip: 4
Video Skip: 1
Information:
Device: Dev2 - PCI-6024E
Rate:4000
Trigger Percent: 100
Trigger Type: Rising Edge
Exact Rate: 4000.0000
Exact Sync Out: 1000.0000
Recorded Rate: 0
Trigger Frame: 0
Data
FFT
2.8
2.6
2.4
2.2
2
1.8
1.6
1.4
1.2
1
0.8
0.6
0.4
0.2
0
-0.2
-0.4
-0.5
-5E+05
-4.5E+05
-4E+05
-3.5E+05
-3E+05
-2.5E+05
-2E+05
-1.5E+05
-1E+05
-50000
11
50000
1E+05
New Cha 1.86523
New Cha 0.00000
New Cha 0.78613
New Cha -0.29297
New Cha 0.41016
New Cha 0.00000
Time: 0.001250 s
Position: 000005
Total Time: 120.000000 s
Total Points: 480000
hold ctrl for pan, shift for zoom
Activate Windows
Go to Settings to activate Windows.

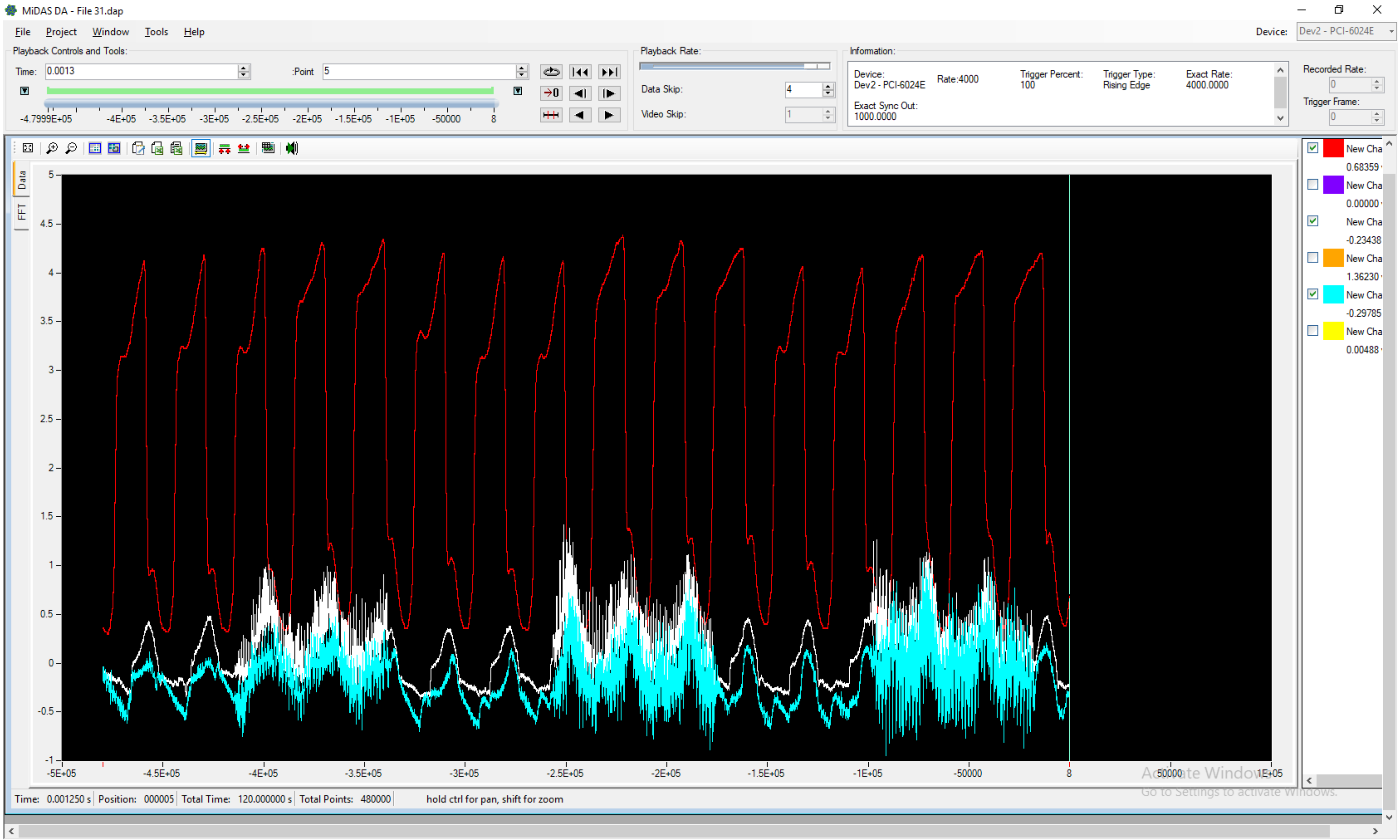
MiDAS DA - File 31.dap
File Project Window Tools Help
Device: Dev2 - PCI-6024E
Playback Controls and Tools:
Time: 0.0013
Point 5
-4.7999E+05 -4E+05 -3.5E+05 -3E+05 -2.5E+05 -2E+05 -1.5E+05 -1E+05 -50000 8
Playback Rate:
Data Skip: 4
Video Skip: 1
Information:
Device: Dev2 - PCI-6024E
Rate:4000
Trigger Percent: 100
Trigger Type: Rising Edge
Exact Rate: 4000.0000
Exact Sync Out: 1000.0000
Recorded Rate: 0
Trigger Frame: 0
Data
FFT
5 4.5 4 3.5 3 2.5 2 1.5 1 0.5 0 -0.5 -1
-5E+05 -4.5E+05 -4E+05 -3.5E+05 -3E+05 -2.5E+05 -2E+05 -1.5E+05 -1E+05 -50000 8 50000 1E+05
New Cha 0.68359
New Cha 0.00000
New Cha -0.23438
New Cha 1.36230
New Cha -0.29785
New Cha 0.00488
Time: 0.001250 s Position: 000005 Total Time: 120.000000 s Total Points: 480000
hold ctrl for pan, shift for zoom
Activate Windows
Go to Settings to activate Windows.
